# Institutional Needs

**Published:** 1914-03-07

**Authors:** 


### Institutional Needs.

"SALUBRA" FOR SANATORIA.

The problem of an economical, non-porous, readily cleaned, and durable wall-covering is an old one to institutional managers, and it is, perhaps, as acutely felt in a sanatorium as in any institution. Samples of "Salubra," which is an oil-paint applied to strong cloth or resistant parchment paper, are well worth a trial on account of the advantages it offers over papers, paints, and enamels of the usual variety. Numerous testimonials from institutional officers remark on its elasticity, by which cracks in the plaster which it covers are hermetically sealed without producing signs of warp or "bubbles" in the "Salubra" itself. Obtainable in various colours, the process by which these are applied under high pressure prevents fading, and the smooth non-porous surface renders practicable the severest scrubbing, which the "Salubra" stands extremely well. Colours which survive hygienic cleansing by disinfectants need no other praise, and as in sanatoria especially such disinfection has to be practised with great regularity, the virtues of "Salubra" have been severely tested in the numerous sanatoria in which it is employed. The makers insist on the freedom from white lead in its manufacture, and state that refined zinc white alone is used. Full specifications may be obtained from the Salubra Company, 18 Old Cavendish Street, W.

